# The relationship between shift work and mental health among electronics workers in South Korea: A cross-sectional study

**DOI:** 10.1371/journal.pone.0188019

**Published:** 2017-11-16

**Authors:** Mo-Yeol Kang, Ho-Jang Kwon, Kyung-Hwa Choi, Chung-Won Kang, Hyunjoo Kim

**Affiliations:** 1 Department of Occupational and Environmental Medicine, Seoul St. Mary’s Hospital, Seoul, Korea; 2 Department of Preventive Medicine, Dankook University College of Medicine, Cheonan, Korea; 3 Taean Environmental Health Center, Taean, Korea; 4 Department of Occupational and Environmental Medicine, Ewha Womans University Mokdong Hospital, Seoul, Korea; Peking University, CHINA

## Abstract

**Objective:**

To determine the relationship between shift work and mental health, particularly insomnia, depression, and suicidal ideation, among electronics production workers.

**Methods:**

A survey was conducted with 14,226 workers from an electronics manufacturer in South Korea. After excluding 112 individuals with incomplete responses, 14,114 respondents were analyzed. As part of a larger project, we collected data on respondents’ general characteristics, work-related characteristics, and health status; however, in this study, we focused on the data related to shift work and mental health. Insomnia, depression, and suicidal ideation were set as dependent variables and working schedule as set as the independent variable. We performed multiple logistic regression analysis with daytime workers as the reference group. The model was adjusted for age, gender, body mass index, workplace, educational level, and marital status with or without children under 6 years of age.

**Results:**

Relative to daytime workers, shift workers had 2.35, 1.23, and 1.17 greater odds of insomnia, depression, and suicidal ideation, respectively. Within the shift worker group, we found that the odds of depression and suicidal ideation increased dramatically when respondents had insomnia. The ORs for depression and suicidal ideation were 4.899 and 7.934, respectively.

**Conclusions:**

Our findings suggest that shift work is related to an increased risk of mental health problems in production workers, and the sleep disturbance related with shift work is a central mechanism for this relationship. Since these results suggest that proactive management of sleep problems might attenuate their detrimental effects on shift worker’s mental health.

## Introduction

Work schedules in the 21^st^ century are longer and more likely to be non-standard than are those in previous centuries, when the workforce operated on strict day–night schedule of working during the day and resting at night. Technological and organizational advances and high employer demands have led to the introduction of shift-work systems to ensure continued operation of various industries. As such, workers in many professions have a schedule that includes work at times other than the daytime. Knutsson (1989) defined shift work ‘‘as an arrangement of working hours that uses teams (shifts) of workers, in order to extend the hours of operation of the work environment beyond that of the conventional office hours” [1].

Shift work is prevalent throughout the world. Approximately 15–20% of employers are estimated to utilize a shift-work system in industrialized countries worldwide. According to data from the Occupational Health Survey (a supplement of the National Health Interview Survey), 29% of all U.S. workers in 2010 worked alternative shifts (i.e., not a regular day shift) and 15% of workers regularly worked a night shift [2]. In Europe, approximately 1 in 5 workers is engaged in any type of shift work that involves night work, and 1 in 10 workers has more than 5 night shifts per month [3]. The Korean Ministry of Employment and Labor examined the work-hour conditions of companies with 10 or more regular employees (n = 3414) in 2011 and found that the around 15.2% of all industries and 22% of all manufacturing companies had a shift system in place [4].

When so many employees are engaged in shift work, it is important determine its possible health-related effects. Shift workers may experience sleep problems when their circadian rhythms promote alertness, and short or poor sleep in turn leads to insufficient recovery and stress [5]. There is also a large and growing body of literature demonstrating the adverse health effects of shift work. A recent review suggested that shift work is related to cardiovascular disease [6], while studies have shown associations between shift work and gastrointestinal complaints, peptic ulcer disease [7], type 2 diabetes [8], and rheumatoid arthritis [9]. Shift work also showed a correlation with cancer, particularly breast cancer [10].

Shift work may also be associated with mental health; however, there is comparatively less research on this issue than on the physical effects, and the results remain inconclusive.

Although our understanding of the impact of shift work on mental health is increasing, most research on this topic has used data from a single or only a few specific occupational groups such as health care professionals [11], especially nurses [12–17], as well as hotel workers [18] and workers in the offshore petroleum industry [19]. There are relatively few studies on workers from the manufacturing industry [20], despite it being a major industry in many countries. Moreover, few studies have addressed the possible gender differences in the effects of shift work on mental health [21].

Within this background, the aim of our study was to determine the relationship between shift work and mental health, in particular insomnia, depression, and suicidal ideation. We investigated this relationship separately for men and women after controlling for certain confounding factors, such as age, marital status, whether children are living at home, education, and workplace.

## Materials and methods

### Data collection and participants

A questionnaire survey was administered to 21,969 workers of an electronic manufacturer in South Korea between April 9 and May 21, 2015. The Institutional Review Board of the Korea National Open University approved this survey (IRB approval number: ABN01-201502-11-02), and all participants gave their written informed consent to participate in the study. Of the 14,241 respondents (response rate 64.8%), 14,104 respondents were analyzed (excluding 122 respondents with incomplete questionnaires).

### Study variables and measurements

Because this study was part of a larger project, we collected considerable information on respondents, including their general characteristics, work-related characteristics, and health status. Trained staff reviewed the completed questionnaires and entered all data into a database. In the present study, we focused on variables related to shift work and mental health.

Respondents were divided into 2 groups according to their work schedule: shift work group and daytime work group. In the workplace from which we recruited respondents, daytime workers worked from 8:00 to 18:00, Monday to Friday, whereas shift workers worked according to a timetable on a 24-day cycle with forward rotation (6 days each of morning, evening, and night shift, with each 6-day block separated by 1–2 days off).

Sleep disturbance was evaluated using the Insomnia Severity Index (ISI), a 7-item self-report questionnaire assessing the nature, severity, and daily life impact of insomnia [22]. The usual recall period is the “past month” and the dimensions evaluated include severity of sleep onset, sleep maintenance, early morning awakening problems, sleep dissatisfaction, interference of sleep difficulties with daytime functioning, noticeability of sleep problems by others, and distress caused by sleep difficulties. Each item was rated on a 5-point Likert scale (0 = no problem, 4 = very severe problem), yielding a total score ranging from 0 to 28. The total score can be interpreted as follows: 0–7, absence of insomnia; 8–14, sub-threshold insomnia; 15–21, moderate insomnia; and 22–28, severe insomnia. In this study, subjects with moderate or severe insomnia were categorized as the insomnia group.

The 4-item screening version of the Center for Epidemiologic Studies Depression Scale (CES-D) was used to evaluate level of depression. The CES-D is a well-known measure of depressive symptoms designed for use in large-sca1e surveys. The short screening version was designed as a brief assessment for broad screening or research purposes [23]. The 4 items are as follows: “I was bothered by things that usually don’t bother me,” “I did not feel like eating, my appetite was poor,” “I felt that I could not shake off the blues even with help of my family or friends,” and “I felt depressed.” Each item is scored on a 4-point Likert scale (0–3), yielding a maximum score of 12 for scales. Higher scores indicate more severe depressive symptoms. A cut-off point of 4 was used to define possible cases of depression. Suicidal ideation was assessed using a single question: “During the past year, have you ever felt like you wanted to die?” Respondents answered this question with “yes” or “no.”

Gender, age, marital status with or without children, education, body mass index (BMI), and workplace were included as covariates for analysis. Marital status was grouped as married, unmarried, and others (including divorce, separation, bereavement, etc.). BMI was calculated as weight/height^2^ (kg/m^2^); a BMI of ≥30 was regarded as obese, and a 30>BMI≥25 was regarded as overweight. Since the surveyed manufacturer comprised two factories located in two different cities, we also included workplace as a covariate.

### Statistical analysis

We initially explored the differences in the demographic variables (age, gender, marital status, children living at home) and prevalence of insomnia, depression, and suicidal ideation between shift-workers and fixed daytime workers using a chi-square test. Then, insomnia, depression, and suicidal ideation were included as dependent variables and working schedule as an independent variable to determine the relationship between shift work and mental health. For this purpose, we used a multiple logistic regression analysis to estimate the multivariate-adjusted odds ratios (ORs) and 95% confidence intervals (95% CIs) for insomnia, depression, and suicidal ideation. Daytime workers served as the reference group. The models were adjusted for age, gender, BMI, workplace, educational level, and marital status with or without children under 6 years of age. As we identified gender differences in the association between shift work and mental health, we undertook further analysis using gender stratification. Furthermore, given that depression and suicidal ideation could be influenced by insomnia, we also estimated the risk of depression and suicidal ideation according to insomnia status in the shift work group, in order to investigate a possible mechanism of depressive symptoms in shift workers. All statistical analysis was performed using SAS version 9.3 (SAS Institute, Cary, NC, USA). A two-tailed alpha of 0.05 was considered significant.

## Results and discussion

### Results

The general characteristics and mental health statuses of our sample are shown in Table 1. Shift workers were significantly younger than were daytime workers; about two-thirds of shift workers were female, while almost four-fifths of daytime workers were male. Approximately 50.8% of shift workers were single, compared with the 35.9% of daytime workers, among whom the married status prevailed (63.2%) to a significant degree (P < 0.0001). About half of Factory A workers and about two-thirds of Factor B workers had a shift-work schedule, but the total number of shift workers was similar between the two factories. Daytime workers had a significantly higher educational level than did shift workers (P<0.0001). Obesity was more prevalent among daytime workers than among shift workers (about 30% vs 20%). Concerning mental health, the prevalence of insomnia among shift workers was nearly thrice that of daytime workers. Likewise, the prevalence rates of depressive symptoms and suicidal ideation were nearly twice as high in shift workers as in daytime workers. All these differences were statistically significant.

**Table 1:** General characteristics and prevalence mental health outcome of study population.

		Working schedule		p-value
		Daytime work	Shiftwork	
		n (%)	n (%)	
N		5635 (40.0)	8469 (60.0)	
Gender				
Male	7243 (51.7)	4395 (78.2)	2848 (33.9)	<0.0001
Female	6776 (48.3)	1226 (21.8)	5550 (66.1)	
Age group				
<30	4688 (33.2)	1146 (20.3)	3542 (41.8)	<0.0001
30-<40	6897 (48.9)	2708 (48.1)	4189 (49.5)	
40-<50	2315 (16.4)	1587 (28.2)	728 (8.6)	
≥50	204 (1.0)	194 (3.4)	10 (0.1)	
Workplace				
Factory A	8568 (61.2)	4001 (71.6)	4567 (54.2)	<0.0001
Factory B	5398 (38.5)	1548 (27.7)	3850 (45.7)	
Others	40 (0.3)	36 (0.6)	4 (0.1)	
Educational level				
High school or less	4749 (33.7)	573 (10.2)	4176 (49.4)	<0.0001
Junior college	4629 (32.9)	910 (16.2)	3719 (44.0)	
College	3609 (25.6)	3080 (85.4)	529 (6.3)	
Graduate school	1093 (7.8)	1061 (18.9)	32 (0.4)	
Marital status				
Unmarried	6323 (44.8)	2016 (35.8)	4307 (50.9)	<0.0001
Married	7567 (53.7)	3563 (63.2)	4004 (47.3)	
Others	214 (1.5)	56 (1.0)	158 (1.9)	
BMI (kg/m^2^)				
<25	10678 (75.7)	3912 (69.4)	6566 (79.9)	<0.0001
25-<30	2939 (20.8)	1523 (27.3)	1393 (16.5)	
≥30	487 (3.5)	185 (3.28)	302 (3.6)	
Insomnia				
Absence	7559 (55.1)	3823 (69.8)	37.36 (45.3)	<0.0001
Sub-threshold	4305 (31.4)	1289 (29.8)	3016 (36.6)	
Moderate	1529 (11.1)	303 (5.5)	1226 (14.9)	
Severe	330 (2.4)	61 (1.1)	269 (3.3)	
Depression*				
No	11972(85.80)	5122(91.91)	6850(81.74)	<0.0001
Yes	1981(14.20)	451(8.09)	1530(18.26)	
Suicidal ideation				
No	13752 (97.2)	5582 (98.6)	8170 (96.2)	<0.0001
Yes	401 (2.8)	77 (1.4)	324 (3.8)	

* Total score of 4-item CES-D is 4 or more

The results of the multiple logistic analyses are shown in Table 2. Relative to daytime workers, shift workers had significantly higher ORs of insomnia, depression, and suicidal ideation. When conducting the stratified analysis by gender, we observed significant gender differences for insomnia and suicidal ideation, but not for depression (insomnia, P for interaction < 0.0001; depression, P for interaction = 0.1044; suicidal ideation, P for interaction < 0.0001). More specifically, the association between shift work and insomnia was stronger in male subjects (male, adjusted OR = 3.314, 95% CI = 2.572–4.270; female, adjusted OR = 1.637, 95% CI = 1.305–2.055). Notably, the associations of depression and suicidal ideation with shift work were only significant among female subjects (Table 2).

**Table 2:** Adjusted ORs and 95% CI values for caseness of insomnia, depressive symptom, and suicidal ideation according to working schedule.

	Working schedule	Insomnia^a^				Depressive symptom^b^				Suicidal ideation			
		n	aOR*	95%CI		n	aOR*	95%CI		n	aOR*	95%CI	
Total	Daytime work	304/5437	1	Reference		441/5653	1	Reference		77/5653	1	Reference	
	Shiftwork	1232/8011	**2.352**	**1.978**	**2.795**	1075/8469	**1.227**	**1.042**	**1.443**	324/8469	**1.186**	**1.183**	**1.189**
Male	Daytime work	205/4395	1	Reference		250/4365	1	Reference		38/4365	1	Reference	
	Shiftwork	398/2848	**3.314**	**2.572**	**4.27**	159/2837	1.004	0.534	1.888	39/2840	1.099	0.824	1.467
Female	Daytime work	154/1226	1	Reference		186/1218	1	Reference		36/1213	1	Reference	
	Shiftwork	1075/5550	**1.637**	**1.305**	**2.055**	902/5528	**1.286**	**1.051**	**1.572**	281/5528	1.246	0.821	1.89

*Adjusted for age, gender, BMI, workplace, educational level, and marital status with or without young children under 6 years of age,

^a^Total score of ISI is 15 or more,

^b^Total score of 4-item CES-D is 4 or more

In the shift worker group, we found that the odds of depression and suicidal ideation increased dramatically when subjects had insomnia. Specifically, the ORs for depression and suicidal ideation were 4.899 and 7.934, respectively, among those with insomnia (Table 3). These results indicate the nonparametric associations between insomnia and the odds of depression and suicidal ideation among shift workers. There were no significant gender differences between the groups.

**Table 3:** Adjusted ORs and 95% CI values for depressive symptoms and suicidal ideation according to insomnia among shift-workers.

		Depressive symptom^a^				Suicidal ideation			
		n	aOR*	95% CI		n(%)	aOR*	95% CI	
Total	Isomnia^b^								
	No	888/6873	1	Reference		166/6974	1	Reference	
	Yes	637/1470	**4.899**	**4.282**	**5.606**	158/1495	**7.934**	**7.909**	**7.959**
Male	Isomnia^b^								
	No	101/2409	1	Reference		22/2436	1	Reference	
	Yes	82/390	**6.128**	**4.432**	**8.475**	17/395	**4.626**	**2.378**	**8.999**
Female	Isomnia^b^								
	No	776/4369	1	Reference		143/4442	1	Reference	
	Yes	541/1050	**4.681**	**4.038**	**5.427**	138/1070	**4.254**	**3.309**	**5.47**

*Adjusted for age, gender, BMI, workplace, educational level, and marital status with or without young children under 6 years of age

^a^Total score of 4-item CES-D is 4 or more,

^b^Total score of ISI is 15 or more

## Discussion

We found that insomnia was significantly more prevalent among shift workers than among daytime workers of an electronics manufacturer in South Korea. Furthermore, shift work had significant associations with depression or suicidal ideation only among female workers. Respondents with insomnia, possibly induced by shift work, also had a much greater odds of having depression or suicidal ideation. Overall, the results suggest that shift work appears to influence workers’ mental health via its aggravation of sleep disturbance.

These results accord with those of other researchers who reported that shift work has significant associations with depression and anxiety. Numerous studies investigating health professionals have shown that nurses, who typically engage in shift work, have a higher prevalence of somatization and anxiety than does the general population [24]. Selvi et al. compared the psychiatric symptoms (using a symptom checklist) of nurses engaged in daytime work with those of nurses engaged in shift work [25]. They found that working night shifts was associated with significantly higher scores on somatization, obsessive–compulsive thoughts, interpersonal sensitivity, anxiety, and paranoid symptoms. A longitudinal study investigating the impact of shift work on the occurrence of a new depressive episode [26] found that men who engage in night work for >4 years have an increased odds of developing an anxiety/depressive disorder (OR = 6.08, 95% CI = 2.06–17.92). There is also opposing evidence from a 10-year observational cohort study, wherein being engaged in present or previous night work was not associated with sickness absence due to mental health problems [20]. Furthermore, one review on shift work concluded that the causal relationships between shift work and mental health are not well established, due to the correlational/cross-sectional design of the majority of studies likewise our study [27]. Hence, further research with longitudinal study design is warranted.

The mechanisms behind the impact of shift work on workers’ mental health may be the disruption of the body’s circadian rhythms, sleep deprivation, or disruption of one’s social life [28]. Night work leads is known to disrupt the body’s circadian rhythms, including the sleep–wake pattern. Several aspects of the endocrine system show changes in secretion patterns over a 24-h period. Most prominently, disrupted endogenous melatonin secretion can cause depression, anxiety, and sleep–wake cycle disorders [29]. Moreover, sleep deprivation and disturbance can lead to depression. Over 18% of shift workers had complaints of insomnia in our study, and these individuals had nearly 5-fold greater odds of depression and 8-fold odds of suicidal ideation than did those without insomnia (Table 3). When we conducted a path analysis, the results showed that the shift work has statistically significant indirect effects on depression and suicidal ideation through insomnia, with about 70% and 65% of the total effect on depression and suicidal ideation, respectively, being mediated by insomnia (see the supplementary material). One recent meta-analysis of 34 cohort studies involving 172,077 participants also suggested a positive relationship between insomnia and depression, with a pooled relative risk (RR) of 2.27 (95% CI: 1.89–2.71) [30].

Besides disturbed sleep and neuroendocrine mechanisms, psychosocial pathways might explain why shift workers experience worse mental health than do fixed daytime workers [31]. Shift work interferes with participation in social networks and family life by leading to desynchronization, given that most familial and social activities are based on the daytime-oriented rhythm of the general population [32].

There were gender differences in the mental health effects of shift work. The stratified analysis by gender showed that the only female respondents with shift work showed significantly greater odds of depression and suicidal ideation. The gender differences in the roles in family life and ability to adapt to shift work might offer an explanation for these differing patterns of mental health effects. Traditionally, women in Asian countries have a greater responsibility for homemaking and childcare than do men. Hence, women might have experienced higher stress levels due to having less time to spend with family during the day. We tried to control for these effects by adjusting for marital status and whether respondents had children living at home. Moreover, male and female workers have different occupations in our sample, which may also explain these observed gender differences in the impact of shift work on mental health. Another possible explanation for the gender differences is that women may be more vulnerable to the adverse effects of shift work because of their more complex circadian and hormonal rhythms [33].

The results of our analysis should be interpreted with some caution because the shift workers included in this study might represent a relatively healthy part of the working population. The respondent selection process is regarded as a critical methodological issue involved in research on shift work. It is possible that the participants in this study had already adapted to their work schedule before the start of the survey. Hence, the survey participants still working in shifts might be considered shift work “survivors,” leading to possibly biased results due to this “healthy shift worker survivor effect.” Therefore, it is possible that the true risks of shift work are underestimated in this cross-sectional analysis. In the future, research on when respondents enter into shift work and day work is needed in order to account for this possible bias in the respondent selection process.

This study has several other limitations. First, the cross-sectional design of the study precludes any causal inferences. Second, the current study data were collected via self-report questionnaires and used only a single question and a simple screener to assess suicidal ideation and depression, respectively. Third, because of the rather low response rate (64.8%) there is some uncertainty as to whether our sample was representative of the population. Finally, all workers were employed at the same company, thus restricting the range in both socioeconomic status and work content; this inhibits the generalizability of the results to the general shift work population.

In conclusion, our findings suggest that shift work is related to an increased risk of mental health problems in electronics production workers, and that there are gender differences in this effect. Our study further indicated that sleep disturbances related to shift work are a central mechanism in this relationship. Since these results suggest that proactive management of sleep problems might attenuate their detrimental effects on worker’s mental health, employers and health managers must pay careful attention to worker’s sleep problems and to develop appropriate intervention strategies in order to prevent mental illnesses in shift workers.

## Supporting information

S1 Table Corrlation analysis. (DOCX)

S1 Fig Path analysis. (DOCX)

S1 File Survey questions used in the study. (DOC)
