# Supplementary material for: The relationship between shift work and mental health among electronics workers in South Korea: A cross-sectional study
Source: PLoS One. 2017 Nov 16;12(11):e0188019. doi: 10.1371/journal.pone.0188019 (PMC5690616; doi:10.1371/journal.pone.0188019)
Supplement: S1 Table — (DOCX) [file pone.0188019.s001.docx]

**S1 Table. Correlation analysis**

| **Correlation analysis** | |  |  |  |  |  |
| --- | --- | --- | --- | --- | --- | --- |
|  |  |  |  |  |  |  |
|  |  | Shiftwork | Insomnia | Depression | Suicidal ideation |  |
|  | Shiftwork | 1 | 0.26546 | 0.18023 | 0.07247 |  |
|  |  |  | <.0001 | <.0001 | <.0001 |  |
|  | Insomnia |  | 1 | 0.48523 | 0.18253 |  |
|  |  |  |  | <.0001 | <.0001 |  |
|  | Depression |  |  | 1 | 0.31033 |  |
|  |  |  |  |  | <.0001 |  |
|  | Suicidal ideation |  |  |  | 1 |  |
|  |  |  |  |  |  |  |
|  |  |  |  |  |  |  |
