# Supplementary material for: The relationship between shift work and mental health among electronics workers in South Korea: A cross-sectional study
Source: PLoS One. 2017 Nov 16;12(11):e0188019. doi: 10.1371/journal.pone.0188019 (PMC5690616; doi:10.1371/journal.pone.0188019)
Supplement: S1 Fig — (DOCX) [file pone.0188019.s002.docx]

**S1 Fig. Path analysis**
