# Supplementary material for: The relationship between shift work and mental health among electronics workers in South Korea: A cross-sectional study
Source: PLoS One. 2017 Nov 16;12(11):e0188019. doi: 10.1371/journal.pone.0188019 (PMC5690616; doi:10.1371/journal.pone.0188019)
Supplement: S1 File — (DOC) [file pone.0188019.s003.doc]

**S1 File. Survey questions used in the study**

**Section A. General characteristics**

1. Where are you currently working?

① Icheon ② Cheongju ③ Others (Seoul, Bundang, etc)

2. Which company are you currently working for?

① SK Hynix ② Others

3. When was the actual birth year?

19 □□ years

4. What is your current height and weight?

1 □□ cm □□□ Kg

-A section is over. The questionnaire continues on the next page.

**Section D. Mental Health**

I. The following is a questionnaire survey to assess the level of stress workers are experiencing. Stress, depression, and suicidal ideation.

1. How often do you feel stress in your daily life?

① I feel very much. ② I feel a lot. ③ I feel a little bit. ④ I rarely feel it.

3. During the past year, have you ever felt like you wanted to die?

① Yes ② No.

II. Below is a list of the ways you might have felt or behaved.

Please tell me how often you have felt this way during the past week.

| During the past week | Rarely or none of the time (less than 1 day ) | Some or a little of the time (1-2 days) | Occasionally or a moderate amount of time (3-4 days) | Most or all of the time (5-7 days) |
| --- | --- | --- | --- | --- |
| 1. I was bothered by things that usually don’t bother me. | ① | ② | ③ | ④ |
| 2. I did not feel like eating, my appetite was poor. | ① | ② | ③ | ④ |
| 3. I felt that I could not shake off the blues even with help of my family or friends. | ① | ② | ③ | ④ |
| 4. I felt depressed. | ① | ② | ③ | ④ |

III. Sleep disorder (Insomnia Severity Index)

In the last two weeks, please indicate how serious each of the following issues are

|  | none | mild | moderate | severe | Very sever |
| --- | --- | --- | --- | --- | --- |
| 1. Difficulty falling asleep. | ① | ② | ③ | ④ | ⑤ |
| 2. Difficulty staying asleep. | ① | ② | ③ | ④ | ⑤ |
| 3. Problems waking up too early. | ① | ② | ③ | ④ | ⑤ |

4. How SATISFIED/DISSATISFIED are you with your CURRENT sleep pattern?

① Very Satisfied ②Satisfied ③Moderately Satisfied ④ Dissatisfied ⑤Very Dissatisfied

5. How NOTICEABLE to others do you think your sleep problem is in terms of impairing the quality of your life? (e.g. daytime fatigue, mood, ability to function at work/daily chores, concentration, memory, mood, etc.)

①Not at all Noticeable ② A Little ③ Somewhat ④Much ⑤ Very Much Noticeable

6. How WORRIED/DISTRESSED are you about your current sleep problem?

①Not at all Worried ② A Little ③ Somewhat ④Much ⑤ Very Much Worried

7. To what extent do you consider your sleep problem to INTERFERE with your daily functioning

①Not at all Interfering ② A Little ③ Somewhat ④Much ⑤ Very Much Interfering

V. Here are some questions about shift work.

1. Have you ever worked shifts?

① Yes. ② No.

If you have experience working in shift work, please fill in the type and period below..

| Factory | Experience | Shift Working period (start) | Shift work period (end) | To date |
| --- | --- | --- | --- | --- |
| 1. 4 crews 3 shifts | □ | □□ year | □□ year | □ |
| 2. 3 crews 3 shifts | □ | □□ year | □□ year | □ |
| 3. 2 crews 2 shifts(24 hours) | □ | □□ year | □□ year | □ |
| 4. 2 crews 2 shifts(12 hours) | □ | □□ year | □□ year | □ |

-D section is over. The questionnaire continues on the next page.

**Section F.** Reproductive Toxicity

1. Do you or your spouse (or sexual partner) have any pregnancy experience? (including marriage or cohabitation

① Yes -> How many times have you been pregnant so far? □ □

-> How many children do you currently have?

-> first birth year □□ year

-> Last year of birth □□ year

② No

**Section A. 인구학적 특성에 관한 질문 (general characteristics)**

1. 현재 근무하고 있는 지역은 어디입니까?

① 이천 ② 청주 ③기타(서울, 분당, 기타)

2. 현재 근무하고 있는 소속사는 어디입니까?

① SK하이닉스 ② 기타

3. 실제 태어난 해는 언제입니까?

19 □□ 년

4. 귀하의 현재 키와 몸무게는 어느 정도입니까?

1□□ cm □□□Kg

-A섹션이 끝났습니다. 다음 페이지에서 설문이 계속됩니다.

**Section D 정신건강**

I. 다음은 근로자들이 평소 겪는 스트레스 정도를 파악하기 위한 설문입니다. 스트레스, 우울 및 자살생각과 관련된 문항으로 구성되어 있습니다.

1. 평소 일상생활 중에 스트레스를 어느 정도 느끼고 있습니까?

① 대단히 많이 느낀다. ② 많이 느끼는 편이다. ③ 조금 느끼는 편이다. ④ 거의 느끼지 않는다.

1. 최근 1년 동안 진지하게 자살을 생각한 적이 있습니까?

① 예 ②아니오.

II. 다음은 기분상태에 관한 질문들입니다.

지난 일주일 동안 아래와 같은 일들이 얼마나 자주 일어났는지 답변해 주십시오.

| 지난 일주일간 나는 | 극히 드물었다.  (1일미만) | 가끔 있었다.  (2~3일) | 종종 있었다.  (3~4일) | 대부분 그랬다.  (5일 이상) |
| --- | --- | --- | --- | --- |
| 1.평소에는 아무렇지도 않던 일들이 괴롭고 귀찮게 느껴졌다. | ① | ② | ③ | ④ |
| 2. 먹고 싶지 않고, 식욕이 없었다. | ① | ② | ③ | ④ |
| 3. 어느 누가 도와준다 하더라도 , 나의 울적한 기분을 떨쳐 버릴 수 없을 것 같았다. | ① | ② | ③ | ④ |
| 4. 상당히 우울했다. | ① | ② | ③ | ④ |

III. 수면장애(불면증지수)

최근 2주간 다음 각 항목의 문제들이 얼마나 심한지 표시해 주세요.

|  | 없음 | 약간 | 중간 | 심함 | 매우 심함 |
| --- | --- | --- | --- | --- | --- |
| 1. 잠들기 어렵다. | ① | ② | ③ | ④ | ⑤ |
| 2. 잠을 유지하기 어렵다. | ① | ② | ③ | ④ | ⑤ |
| 3. 쉽게 깬다. | ① | ② | ③ | ④ | ⑤ |

4. 현재 수면양상에 대해서 얼마나 만족하고 있습니까?

① 매우 만족 ② 약간 만족 ③ 그저 그렇다. ④ 약간 불만족 ⑤ 매우 불만족

5. 귀하의 수면장애가 어느 정도나 당신의 낮 활동을 방해한다고 생각하십니까?

(낮에 피곤함, 직장이나 가사에 일하는 능력, 집중력, 기억력, 기분 등)

① 전혀 방해되지 않는다. ② 약간 ③ 다소 ④ 상당히 ⑤ 매우 많이

6. 주변 사람들이 귀하가 수면문제로 삶의 질이 떨어지고 있다고 여기고 있습니까?

① 전혀 그렇지 않다. ② 약간 ③ 다소 ④ 상당히 ⑤ 매우 많이

7. 당신은 현재 수면 문제에 관하여 얼마나 걱정하고 있습니까?

① 전혀 걱정하지 않는다. ② 약간 ③ 다소 ④ 상당히 ⑤ 매우 많이

V. 다음은 교대근무에 관한 질문들입니다.

1. 귀하께서는 교대근무를 하신적이 있습니까?

① 있다. ②없다.

교대근무 경험이 있다면 아래 종류와 기간을 기입해 주십시오.

|  | 경험 | 교대근무기간(시작) | 교대근무기간(종료) | 현재까지 |
| --- | --- | --- | --- | --- |
| 1. 4조 3교대 | □ | □□년 | □□년 | □ |
| 2. 3조 3교대 | □ | □□년 | □□년 | □ |
| 3. 1일 맞교대 | □ | □□년 | □□년 | □ |
| 4. 주야간 맞교대 | □ | □□년 | □□년 | □ |

D 섹션이 끝났습니다. 다음 페이지에서 설문이 계속됩니다.

**Section F. 생식독성**

1. 귀하 또는 귀하 배우자(혹은 성관계 상대)께서는 임신 경험이 있습니까?( 사실혼, 동거 모두 포함)

① 예 ->현재까지 총 몇 번이나 임신 경험이 있습니까?□번

->현재 귀하의 자녀는 몇 명입니까?□번

-> 첫 출산년도 □□년

->마지막 출산년도 □□년

② 아니오
